# Case Report: Use of Amniotic Membrane for Tectonic Repair of Peripheral Ulcerative Keratitis With Corneal Perforation

**DOI:** 10.3389/fmed.2022.836873

**Published:** 2022-04-27

**Authors:** Maryam Eslami, Blanca Benito-Pascual, Saadiah Goolam, Tanya Trinh, Greg Moloney

**Affiliations:** ^1^Department of Ophthalmology and Visual Sciences, University of British Columbia, Vancouver, BC, Canada; ^2^Sydney Eye Hospital, Sydney, NSW, Australia; ^3^Save Sight Institute, University of Sydney, Sydney, NSW, Australia; ^4^Mosman Eye Centre and Narellan Eye Specialists, Sydney, NSW, Australia

**Keywords:** amniotic membrane transplantation, corneal perforation, peripheral ulcerative keratitis, corneal ulceration, tectonic graft repair

## Abstract

**Purpose:**

To provide a perspective and surgical video demonstration of peripheral corneal ulceration and perforation managed with multilayered amniotic membrane transplantation.

**Case Reports:**

Case 1 describes a 48-year-old female with progressive redness and pain, and an inferonasal corneal thinning and perforation in the left eye from peripheral ulcerative keratitis. She underwent conjunctival recession with amniotic membrane inlay and onlay (Sandwich technique) transplantation. The amniotic membrane integrated well, and her Snellen visual acuity improved from 6/21 preoperatively to 6/9 at 3 months post op. Case 2 describes a 78-year-old male with redness and pain with temporal corneal thinning bilaterally and perforation in the right eye from peripheral ulcerative keratitis. Both eyes underwent similar surgical intervention with smooth integration of the amniotic membrane in the cornea and improvement in the visual acuity. Both patients were also started on systemic immunosuppression in collaboration with the rheumatology team.

**Conclusion:**

We report successful use of multilayered amniotic membrane transplantation for the treatment of corneal ulceration and perforation. The authors believe the simplicity of the surgical technique, easier access to amniotic membrane tissue, and lower induced post-operative astigmatism all provide advantages over alternative treatment modalities.

## Introduction

Various corneal pathologies can lead to corneal perforation, including infectious keratitis (bacterial, viral, fungal or parasitic), inflammatory keratitis (Mooren's ulcer, rheumatoid arthritis, systemic lupus erythematosus), neurotrophic keratitis, peripheral corneal thinning (pellucid marginal degeneration, Terrien's marginal degeneration), trauma, and chemical injuries (1–3).

The management of corneal perforation varies from non-surgical treatments such as bandage contact lenses or tissue adhesives, to surgical modalities like corneal suturing, conjunctival flaps, amniotic membrane (AM) transplantation and ultimately tectonic corneal patch graft (2, 3). The treatment chosen often depends on the size, location, and etiology of the corneal perforation, as well as the surgeon's experience and availability of donor tissues (amniotic membrane or donor cornea) (3, 4).

AM may be used as a graft (inlay), patch (onlay) or both for the management of corneal ulcers and perforations (5). It is not immunogenic, prevents apoptosis and has antimicrobial, antifibrotic, anti-inflammatory and antiangiogenic properties (3, 6). AM enhances epithelialisation by facilitating migration and differentiation of epithelial cells, reinforcing adhesion of basal epithelial cells, and regulating proliferation of normal corneal, conjunctival, and limbal fibroblasts (3).

In this paper, we present two cases of corneal perforation secondary to peripheral ulcerative keratitis managed with a sandwich technique of AM transplantation demonstrated in the Supplementary Video. Informed consent was obtained from both patients for publication of this case report.

## Case 1

A 48-year-old female presented with progressive pain, redness, and foreign body sensation in the left eye (LE) over the past 6 months. She had a history of peripheral ulcerative keratitis in the right eye (RE) requiring systemic immunosuppression and tectonic lamellar keratoplasty to reconstruct the area of the corneal thinning 15 years ago. Her systemic workup (complete cell blood count, electrolytes, urea, creatinine, liver function test, thyroid function test, C reactive protein (CRP), erythrocyte sedimentation rate (ESR), rheumatoid factor, antinuclear antibodies (ANA), antineutrophil cytoplasmic antibodies (ANCA), extractable nuclear antigens, anti-citrullinated protein antibody, syphilis, herpetic and hepatitis serologies) was negative 15 years prior. Her systems review at that point revealed a chronically inflamed elbow; therefore, she was started on oral cyclosporine up to 100 mg oral BID and Felodipine 2.5 mg oral daily for presumed seronegative rheumatoid arthritis. This systemic management was successfully tapered off 4 years ago with no recurrence of symptoms in the eye or the elbow joint.

On examination, she had uncorrected Snellen visual acuity of 6/30 on the right and 6/21 on the left. RE revealed an uninflamed tectonic lamellar keratoplasty. The LE was acutely inflamed inferonasally adjacent to an area of peripheral corneal ulceration without perforation. The anterior chamber was deep and quiet bilaterally at this point. She had trace nuclear sclerosis in both eyes.

**Figure 1:**
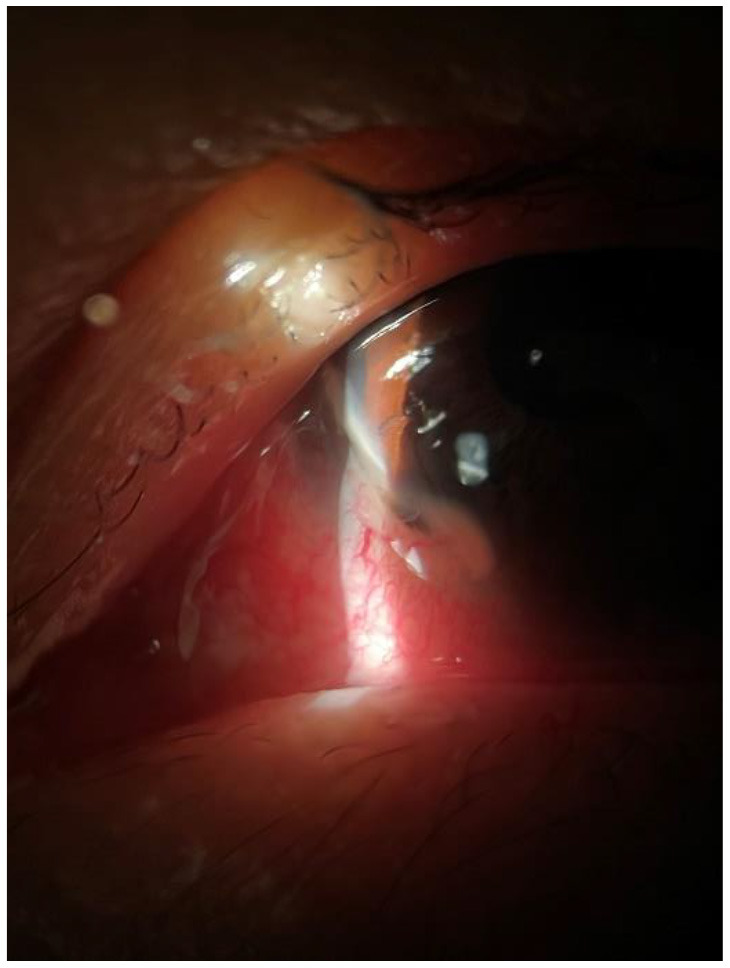
Inferonasal corneal ulceration with adjacent inflammation of the left eye.

She was commenced on oral ascorbic acid, topical moxifloxacin QID andprednisolone acetate 1% QID in the LE, and advised to use an eye shield at night. The rheumatology team was consulted again, and she was started on oral prednisone 60 mg daily as well as oral mycophenolate mofetil 1 g BID. On repeat assessment a week later, she had not yet started systemic treatment and had not been using her eye shield. She was found to have a perforation within the area of thinning with a flat anterior chamber (Figure 1).

A bandage contact lens was put on the LE as a temporizing measure before surgical management. She was continued on topical moxifloxacin and prednisolone acetate in the LE. Two days later, a conjunctival recession, amniotic membrane transplant and a temporary tarsorrhaphy was performed under retrobulbar anesthesia. A single piece of folded, multi-layered, fresh frozen amniotic membrane was packed and sutured into the LE corneal defect and held in place using fibrin glue. An overlying large single layer of amniotic membrane with the epithelial side down was sutured with interrupted sutures of 10.0 nylon with tension over the temporal ocular surface in a bandage fashion (Supplementary Video). A medial temporary tarsorrhaphy was carried out using bolsters and 6-0 nylon. Subconjunctival antibiotics of cefazolin and dexamethasone were injected at the end of the procedure. Postoperatively the patient was commenced on moxifloxacin eye drops QID, prednisolone acetate 1% QID and preservative-free lubricants 2-hourly.

**Figure 2:**
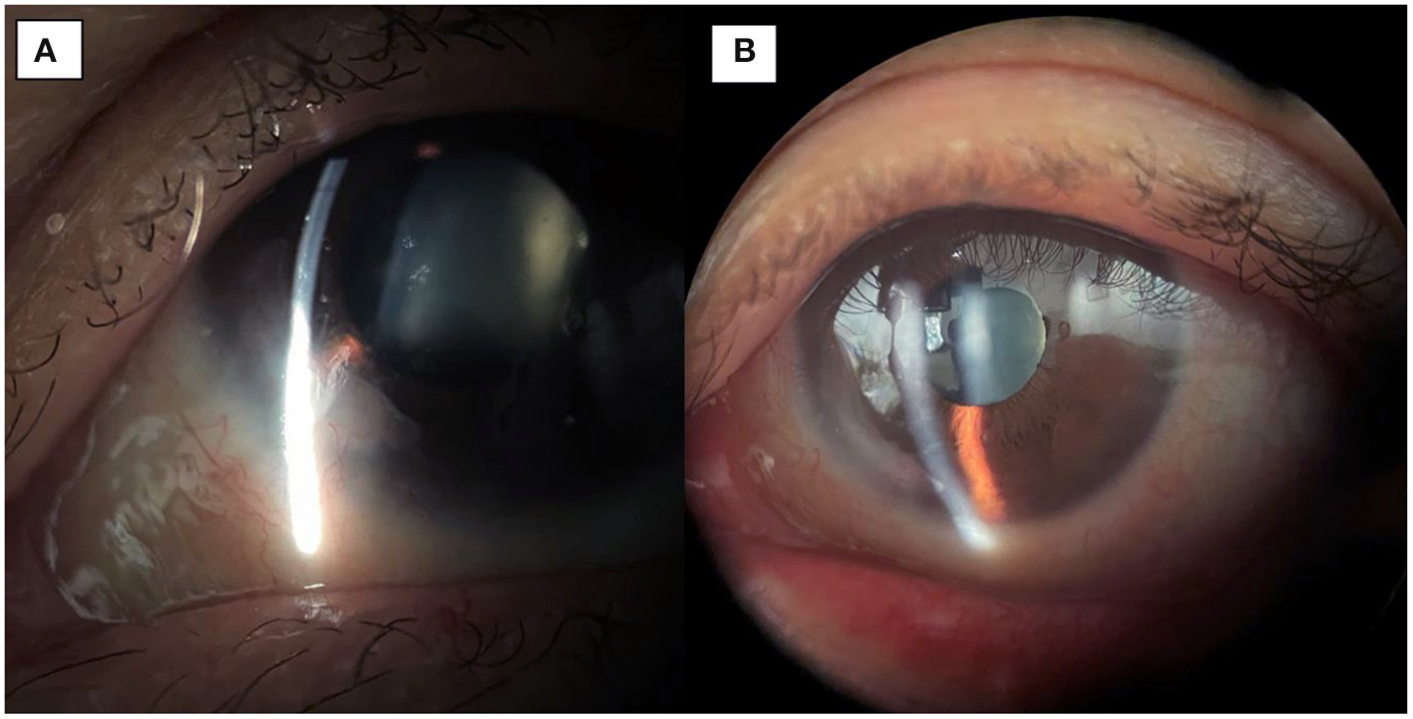
**(A)** 1-month post-op image showing integration of the amniotic membrane patch in the inferonasal corneal stroma. **(B)** 3-month post-op image demonstrating smooth integration and an uninflamed left eye.

The patient had an uneventful post-operative course without further episodes of ulceration, melt or inflammation. The AM patch integrated well into the corneal stroma at the 1-month postoperative visit with Snellen visual acuity of 6/15 and a quiet ocular surface (Figure 2A). Her uncorrected Snellen visual acuity improved to 6/9 at 3-months post op (Figure 2B).

## Case 2

A 78-year-old male from rural New South Wales presented to the Sydney Eye Hospital emergency department with a RE corneal perforation following a 4-day history of severe right ocular pain.

Past ocular history included LE pseudophakia and bilateral pterygium excision several years ago as well as bilateral dry age-related macular degeneration. He had a medical history of hypertension and ischemic heart disease with no other systemic complaints.

**Figure 3:**
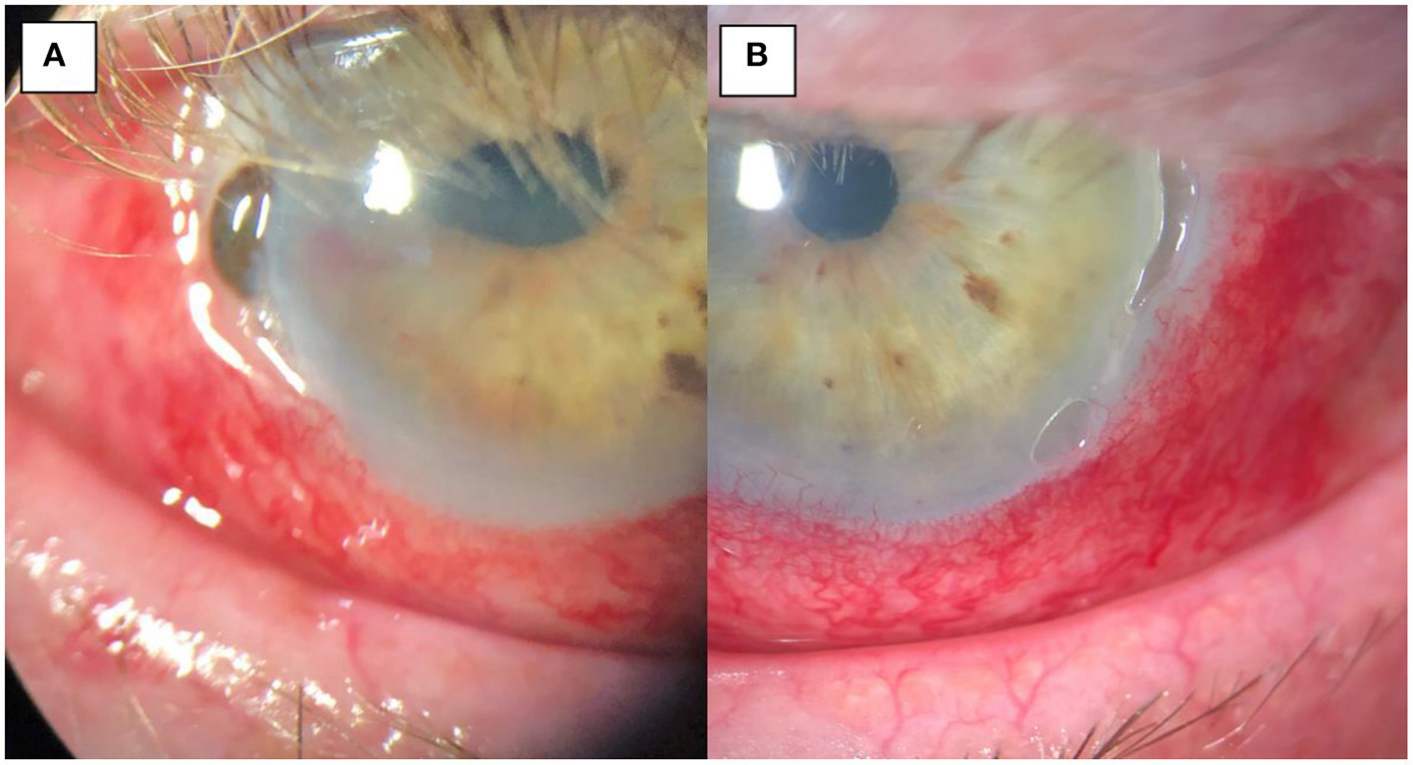
**(A)** Pre-operative image of the right eye with temporal thinning and perforation, iris prolapse and surrounding inflammation. **(B)** pre-operative image of the left eye with temporal thinning and surrounding inflammation but no corneal perforation.

Examination on presentation revealed Snellen visual acuities of 6/38 in the RE and 6/24 in the LE. Intraocular pressure (IOP) was 13 and 11 mmHg in the right and left eye, respectively. Conjunctiva were bilaterally injected without any scleritic foci or evidence of scleromalacia. Corneas bilaterally were found to have temporal peripheral ulcerative keratitis (PUK) (6.5 mm in the RE and 7 mm in the LE), with RE corneal perforation of 2 mm with iris prolapse and LE thinning of ~80–90% within the area of ulceration (Figures 3A,B). RE Anterior chamber was almost flat with 3+ cells and a fibrinous reaction. LE anterior chamber was deep with 2+ cells. Other examination findings included pseudoexfoliative cataract in the RE and pseudophakia in the LE.

A cautious corneal scrape and swab of the RE ulcer bed excluded a superimposed microbial keratitis. Blood tests mentioned above, quantiferon gold test and a chest X-Ray were ordered to exclude infective and inflammatory/autoimmune causes of peripheral ulcerative keratitis. Blood tests were positive for a raised ESR (18 mm/h) and ANA (1:320 speckled); the remaining blood tests were unremarkable.

The patient was admitted on an initial treatment regimen of fortified topical antibiotics (gentamicin 0.9% and Cefazolin 5%) hourly in the RE and QID in the LE, topical prednisolone sodium phosphate preservative free 0.5% twice a day in both eyes, oral anti-collagenolytic agents (ascorbic acid 2 grams daily, doxycycline 100 mg twice a day), oral prednisolone 60 mg daily, oral ciprofloxacin 750 mg twice a day and valacyclovir 500 mg three times a day.

Gluing of the corneal perforation as a temporizing measure was not possible due to the significant area of perforation and degree of iris prolapse. For the RE, a conjunctival recession, amniotic membrane transplants and tarsorrhaphy was performed. A single piece of folded, multi-layered, fresh frozen amniotic membrane was packed and sutured into the RE corneal defect and an overlying large single layer of amniotic membrane with the epithelial side down was sutured with tension over the temporal ocular surface with interrupted sutures of 10.0 nylon. A nasal paracentesis was used to reform the anterior chamber and reposit the iris. A second layer of amniotic membrane was then applied to the entire ocular surface with a purse-string suture.

The LE had conjunctival recession and 3 glue patches applied to the area of melt before a layer of amniotic membrane was glued over the area of thinning incorporating the area of conjunctival recession. Subconjunctival antibiotics of cefazolin and dexamethasone were injected bilaterally at the end of the procedures.

Postoperatively the patient was commenced on preservative-free chloramphenicol 0.5% drops QID, cyclosporine 1% BID and preservative-free lubricants 2-hourly. Topical steroids and fortified antibiotics were ceased. The rheumatology team was consulted as part of the multidisciplinary management of this patient's idiopathic immune-mediated corneal disease. 3 cycles of intravenous methyl prednisolone 500 mg and intravenous cyclophosphamide 650 mg were administered, followed by a tapering dose of the oral prednisone and initiation of long-term immunosuppression with oral mycophenolate 360 mg twice daily.

**Figure 4:**
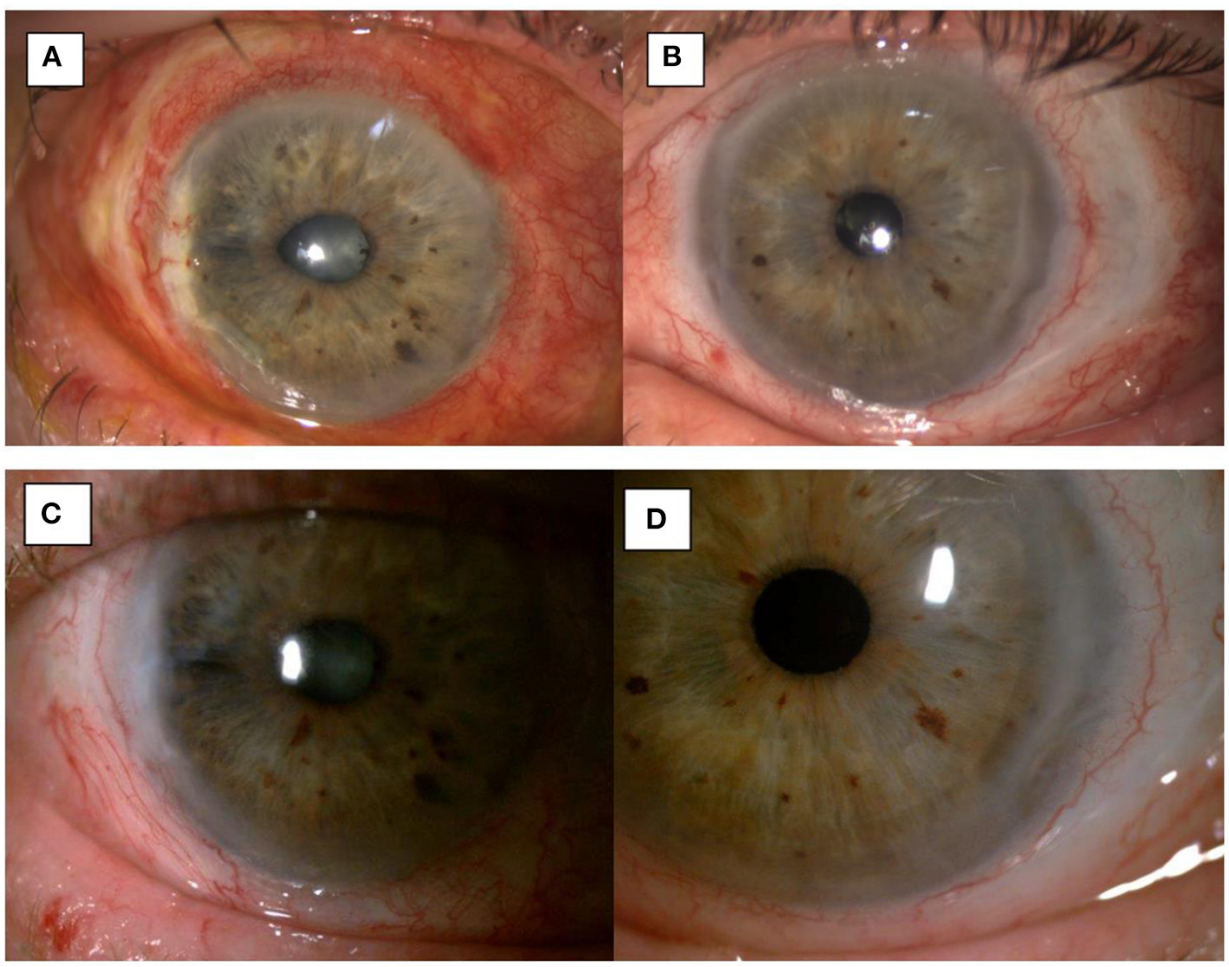
1-month post-op image showing integration of the amniotic membrane patch in the temporal corneal stroma of the right eye **(A)** and the left eye **(B)**. 3-month post-op image showing uninflamed eyes and a smooth integration of the amniotic membrane patch in the temporal corneal stroma of the right eye **(C)** and the left eye **(D)**.

The patient had an uneventful post-operative course without further episodes of ulceration, melt or inflammation. Snellen visual acuity at the 1-month postoperative visit was 6/90 in RE (with cataract) and 6/9 in LE with normal intraocular pressures and a quiet ocular surface (Figures 4A,B).

Topical and systemic immunosuppressants were reduced as the patient continued on a stable postoperative course. At the 4-month post-operative visit the patient had similar visual acuity, and was maintained on topical preservative free lubricants and Mycophenolate 500 mg BD (Figures 4C,D).

## Discussion

In this article, we present two cases of peripheral ulcerative keratitis (3 eyes) with corneal perforation treated with multi-layered amniotic membrane transplantation using cigar technique demonstrated in the Supplementary Video. We found the AM integrated well into the host cornea in all 3 eyes with rapid visual recovery in all eyes but one, in which the reduced vision was attributed to cataract formation.

To replace the corneal tissue defect and fill in the corneal ulceration, the main options are fibrin glue, conjunctival and tenons tissue, donor corneal tissue, and AM (6–8). Fibrin glue, although a valuable tool in small perforations, is often ineffective on its own in filling the entire depth of corneal ulceration in large defects and may prolong wound healing and closure of epithelial defect (7). Conjunctival tissue often leads to neovascularization, scarring and conjunctivilization of the epithelium (7). Donor corneal tissue is often in short supply or may not be immediately available for the treatment of corneal perforation (7). It also has an increased risk of rejection and ongoing melt due to the inflamed host cornea and the underlying rheumatic disease (8). Many studies have reported on the successful use of AM in the treatment of corneal perforation (1–4, 6, 7). Some authors believe AM transplantation to be superior to alternatives due to AM's antifibrotic, anti-inflammatory and antiangiogenic properties that not only fills the defect and restores the globe's integrity, but also prevents further tissue loss (3, 6, 7). This technique does require a tightly “packed” scroll of AM into a “cigar” shape—we find that this contour lends itself well to simultaneously filling in the central “bulk” for the largest portion of the defect alongside tapered “ends” and each pole of the “cigar”, allowing for a gradual filling of crescenteric defects at superior and inferior ends. The initial placement of sutures from the host-AM-host requires replacing with tighter tension as the walls of the crevice become more and more closely apposed.

**Figure 5:**
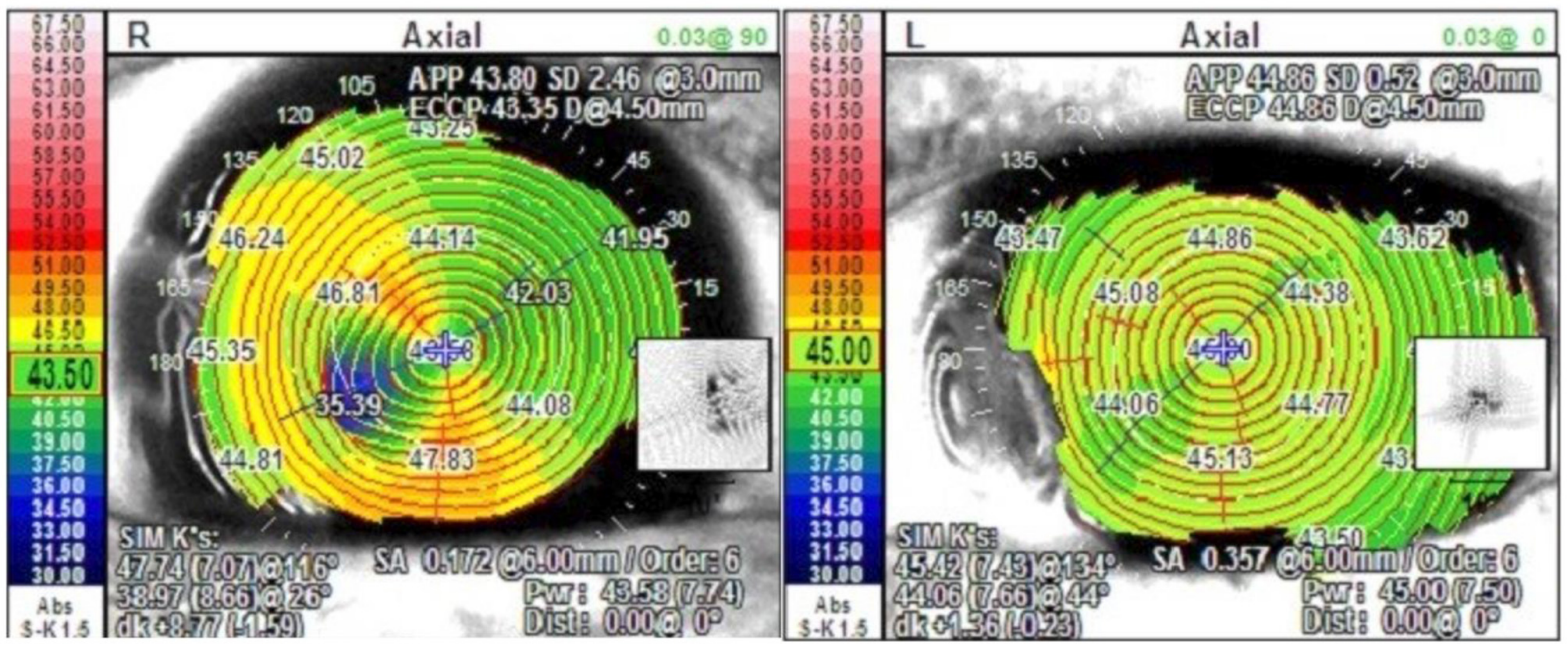
Topographic map of Case 1 post operatively with tectonic corneal graft in the right eye and AM transplantation in the left eye.

In our cases, we found the rapid visual rehabilitation and low induced astigmatism to be superior to tectonic corneal graft as well. The first case previously required a tectonic lamellar keratoplasty in the right eye for the same indication with an induced astigmatism of 6.5 diopters, while the left eye of the same patient treated with AM transplantation described above had a cylinder of 0.75 diopters at 3 months (Figure 5). This is consistent with prior studies with some authors reporting declining astigmatism with time as the AM continues to integrate into the host cornea (1, 3).

Lastly, the simplicity of the surgical technique (Supplementary Video) and the relative ease of availability of AM compared to donor cornea makes this an attractive choice for the management of corneal ulcers and perforations. This was also highlighted in a study by Ngan and Chau (9) on Mooren ulcers in Vietnam where systemic immunosuppressive medications are not readily available. Indeed, the management of corneal melting and perforation can be complex, and the treatment modality chosen may vary significantly depending on the clinical picture, other ocular comorbidities, and the size, location, and etiology of corneal thinning (Table 1). The authors agree that AM transplantation may not be suitable as the sole treatment of central corneal ulceration, as it leads to corneal opacity once healed and may degrade visual acuity. However, it may be an appropriate temporizing measure to allow for an uninflamed and quiet eye prior to donor corneal graft transplantation, thereby increasing success for visual rehabilitation. Additionally, while there is no absolute perforation size cut-off for the use of AM transplantation, it may be difficult to pack and close large perforation defects and restore the globe's integrity. However, its successful use in large or 360 degrees of corneal thinning has been previously reported (9).

**Table 1:** A comparison of advantages and preferred utilization of AM transplantation and tectonic corneal graft.

	**AM transplantation**	**Tectonic corneal graft**
Advantages:	• Rapid recovery • Relative availability and ease of access • Relative simplicity of surgical technique • Less induced astigmatism • Less risk of rejection and ongoing melt	• Transparent tissue • Superior structural integrity with less potential for dislodgement
The technique may be preferred in:	• Peripheral corneal melt • Large areas of thinning with small or medium-sized perforation • Inflamed eye • Rheumatic etiology of corneal melt	• Large-sized perforation • Central disease • Intraocular tissue involvement

## Conclusion

Multi-layered amniotic membrane transplantation may be an effective surgical modality in the treatment of corneal ulceration and small to mid-sized perforation from peripheral ulcerative keratitis. The surgical technique is simple and leads to relatively rapid visual recovery with low induced astigmatism.

## Ethics Statement

Ethical review and approval was not required for the study on human participants in accordance with the local legislation and institutional requirements. The patients/participants provided their written informed consent to participate in this study. Written informed consent was obtained from the individual(s) for the publication of any potentially identifiable images or data included in this article.

## Author Contributions

ME, BB-P, and SG drafted the manuscript and the two cases. TT and GM were supervisors, performed the surgeries, and edited the manuscript and the surgical video. All authors contributed to the article and approved the submitted version.

## Conflict of Interest

The authors declare that the research was conducted in the absence of any commercial or financial relationships that could be construed as a potential conflict of interest.

## Publisher's Note

All claims expressed in this article are solely those of the authors and do not necessarily represent those of their affiliated organizations, or those of the publisher, the editors and the reviewers. Any product that may be evaluated in this article, or claim that may be made by its manufacturer, is not guaranteed or endorsed by the publisher.
